# Violence toward physicians in emergency departments of Morocco: prevalence, predictive factors, and psychological impact

**DOI:** 10.1186/1745-6673-5-27

**Published:** 2010-09-28

**Authors:** Jihane Belayachi, Kamal Berrechid, Fatiha Amlaiky, Aicha Zekraoui, Redouane Abouqal

**Affiliations:** 1 Medical Emergency Department, Ibn Sina University Hospital, 10000, Rabat, Morocco; 2 Laboratory of Biostatistics, Clincial and Epidemiological Research, Faculté de Médecine et Pharmacie - Université Mohamed V, 10000, Rabat, Morocco

## Abstract

**Introduction:**

Anyone working in the hospital may become a victim of violence. The effects of violence can range in intensity and include the following: minor physical injuries, serious physical injuries, temporary or permanent physical disability, psychological trauma, and death. The aim of this study was to determine the frequency of exposure, characteristics, and psychological impact of violence toward hospital-based emergency physicians in Morocco.

**Methods:**

This was a survey including emergency physicians who ensured emergency service during the last fortnight. The variables studied were those related to the victim (age and gender), and those related to aggression: assaulter gender, number, time, reason (delay of consultation and/or care, acute drunkenness, neuropsychiatric disease), and type (verbal abuse, verbal threat and/or physical assault). After the questionnaire was completed, State-Trait Anxiety Inventory (STAI) of Spielberg was applied to all participants.

**Results:**

A total of 60 physicians have achieved permanence in emergency department during the 15 days preceding the questionnaire response. The mean age was 24 ± 1 year and 57% were male. A total of 42 (70%) had been exposed to violence. The violence occurred at night n = 16 (27%), afternoon n = 13 (22%), evening n = 7 (12%) and morning n = 6 (10%). Reasons for violence were: the delay of consultation or care in n = 31 (52%) cases, acute drunkenness in n = 10 (17%) cases and neuropsychiatric disease in n = 3 (5%) cases. Twenty eight (47%) participants stated that they experienced verbal abuse, n = 18 (30%) verbal threat and n = 5 (8.3%) physical assault. Exposure to some form of violence was related to a higher median [interquartile range, IQR] state anxiety point (SAP); (51 [46-59] vs 39 [34-46]; *P *< 0,001), and trait anxiety point (TAP) (48 [41-55] vs 40,5 [38-53]; *P *= 0,01).

**Conclusions:**

This study revealed a high prevalence (70%) of violence toward doctors in Morocco emergency departments. The exposure of physicians to some form of violence is greater among doctors with anxiety trait and was related to significant degree of anxiety state.

## Introduction

The terms "workplace aggression" and "workplace violence" are often used interchangeably, they are distinguishable. Schat & Kelloway suggested that workplace violence is a distinct form of workplace aggression. It comprises behaviours that are intended to cause physical harm (physical assaults and/or the threat of assault) [1]. All violent behaviours are aggressive whereas not all aggressive behaviours are violent [2]. Workplace violence was similar to workplace aggression, but the behaviour usually is more physical in nature. Schat & Kelloway offered a general definition of workplace aggression as "behaviour by an individual or individuals within or outside an organization that is intended to physically or psychologically harm a worker or workers and occurs in a work-related context."[1]. They suggested that this definition (*a*) was consistent with definitions used in the general human aggression literature [3,4], (*b*) was sufficiently general to include a wide range of physical and nonphysical behaviours that comprise workplace aggression, and (*c*) encompassed aggressive behaviours enacted by a variety of sources within (supervisors, co-workers) and outside (clients, customers, patients) of the organization [5,6]. Workplace violence has become an alarming phenomenon worldwide. The real magnitude of the problem is largely unknown, and recent information shows that the current knowledge is only the tip of the iceberg [7]. While workplace violence affects practically all sectors and all categories of workers, the health sector is at major risk. Violence in this sector may constitute almost a quarter of all violence at work [7]. Violence in the emergency department is a common concern as well [8,9]. It appears that emergency department staff work in an environment where they are constantly exposed to situations with aggressive individuals [9]. Although anyone working in a hospital may become a victim of violence, physicians who have the most direct contact with patients are at higher risk. The effects of violence can range in intensity and include the following: minor physical injuries, serious physical injuries, temporary or permanent physical disability, psychological trauma, and death. Violence may also have negative organizational outcomes in the form of low worker morale, increased job stress, increased worker turnover, reduced trust of management and co-workers, and a hostile working environment [10]. To our knowledge, this is the first study to evaluate prevalence and impact of violence in the emergency departments in Morocco. The aim of this study was to determine the frequency of exposure to violence, characteristics, and psychological impact of violence toward hospital-based physicians in emergency departments.

## Methods

### Study design and setting

This was a survey of emergency physicians who ensured service in emergency departments. Ibn Sina University hospital in Rabat is a referral centre for habitants of Western-North Morocco, it is a 1028 bed tertiary - stage hospital that opened in 1955. The bed occupancy rate is of 76% to 85%. The hospital comprises 24 departments (12 surgical, 9 medicals, and 3 intensive care units). Gynecology-Obstetric and pediatric patients are treated in other institutions. The mean emergency department visits per day is 176. The emergency department comprises 2 units (medical and surgical unit), this department is staffed 24 hours a day by intensive care physicians and with a complement of rotating residents. These are students who have finished medical studies and have won a competition to become resident in University Hospital. They work in the emergency service, and in parallel, conduct training in medicine, surgery, pediatrics and obstetrics-gynecology units. All physicians who ensured emergency services during the 15 days preceding the survey were included.

### Data collection and definitions

We defined workplace aggression as "behaviour by an individual or individuals within or outside an organization that is intended to physically or psychologically harm a worker or workers and occurs in a work-related context" [1].

We surveyed emergency physicians who ensured emergency service during the last fortnight. Physicians were approached individually by trained research assistants. They explained the purpose of the study, distributed the survey in hard copy form, and invited them to complete a questionnaire. The questionnaire was recovered after completion and was returned by hand to an investigator, with all information being anonymous and confidential. There was therefore no requirement for ethical approval. The survey questionnaire included the characteristics related to the victim (age and gender), and those related to the violence: the time, the reason (delay of consultation and/or care, acute drunkenness, neuropsychiatric disease), and the kind of assault (verbal abuse, verbal threat and physical assault), and with respect to the physical assault, which device was used. Delay of consultation is the waiting time before consultation. Waiting time is usually defined by the duration from the time a patient registered in the emergency department to the time they were seen by a doctor [11]. Delay of care included processing time that was defined as the duration from registration to leaving emergency department, which included discharge home, admission to hospital, admission to the observation ward, or certification of death [11]. Delay of consultation or care is the reason reported by patient or his family. It is the time perceived by the patient or his family. No real limit has been previously established.

Aggression, raising of voices (screaming) and name calling were defined as verbal abuse. The raising of fists and attempts at physical violence were defined as verbal threats (aggressor does not touch the victim but attempts to physically assault). Slapping, kicking, throwing any item or object, biting, hitting, slapping, pulling, pushing, pinching, grabbing, scratching and punching were defined as physical assault. After the questionnaire was completed, State - Trait Anxiety Inventory (STAI) of Spielberger was applied to all participants. It contains 40 multiple-choice questions written on a 4-point Likert scale, classified as always, often, sometimes, and rarely. The score ranges between 20 and 80 points for each scale. It is a self-report assessment device; it can be completed in ten minutes or less; which includes separate measures of State and Trait Anxiety. Each measure is divided into five indices: very low (≤ 35), low (36-45), medium (46-55), high (56-65) and very high (≥ 66). The essential qualities evaluated by the STAI scale are feelings of apprehension, tension, nervousness, and worry [12]. Scores on the STAI scale increase in response to psychological stress, and decrease as a result of relaxation training [12]. State and trait anxiety are defined by Spielberger as follows; State anxiety is defined as an unpleasant emotional arousal in face of threatening demands or dangers. Trait anxiety, on the other hand, reflects the existence of stable individual differences in the tendency to respond with state anxiety in the anticipation of threatening situations [12].

### Statistical analyses

Data are presented as mean ± standard deviation for variables with a normal distribution, and as median and interquartile range for variables with skewed distributions. Parametric or nonparametric tests were used for continuous variables as appropriate after the normality of the distribution was tested by the Kolmogorov-Smirnov test with Lilliefors correction. Statistical differences between groups were evaluated by the chi-square test for categorical variables. Comparison of group differences for continuous variables was carried out by Student-test or the Mann-Whitney U-test. A two-tailed *P *value < 0.05 was considered significant. Statistical analyses were carried out using SPSS for Windows (SPSS, Inc., Chicago, IL, USA). Internal consistency reliability of the French version of STAI was assessed using Cronbach's coefficient alpha; a high alpha coefficient (≥ 0.70) suggests that the items within a scale measure the same construct and support the construct validity [13].

## Results

### Violence characteristics

A total of 60 physicians achieved permanence in the emergency departments during the 15 days preceding the questionnaire response. The mean age of the study participants were 24 ± 1 year and 57% were male. A total of 42 (70%) had been exposed to a form of violence, of which 19(45%) were women, and 23 (55%) were men. Twenty eight (47%) participants stated that they experienced verbal abuse, 30% (n = 18) verbal threat and 8.3% (n = 5) physical assault. The violence occurred at night n = 16 (27%), afternoon n = 13 (22%), evening n = 7 (12%), and morning n = 6 (10%). Reasons for violence were: a delay of consultation or care in 31(52%) cases, acute drunkenness in 10 (17%) cases and neuropsychiatric disease in 3(5%) cases. Table 1 shows the characteristics of violence.

**Table 1:** Characteristics of violence

Variables	
**Victims**	
Age, years, median [IQR]	24 [23-25]
Mal gender, n (%)	23 (55%)
**Characteristics of aggression, n (%)**	
Time	
Morning	6 (10%)
Afternoon	13 (22%)
Evening	7 (12%)
Night	16 (27%)
Reason	
Delay of consultation or care	31 (52%)
Acute drukenness	10 (17%)
Neuropsychiatrics disease	3 (5%)
Kind	
Verbal abuse	28 (48%)
Verbal threat	18 (30%)
Physical assault	5 (3.3%)

Data are expressed as median (interquartile range: IQR) or as number (percentage).

### State - Trait Anxiety Inventory results

**Figure 1:**
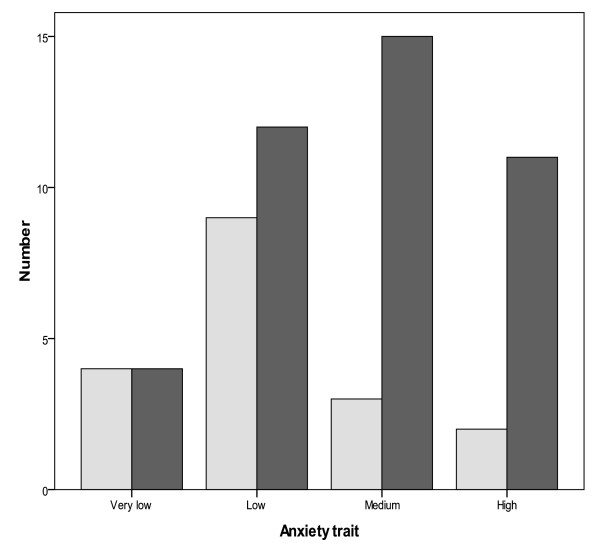
**Comparison of indices of trait anxiety inventory between assaulted (shaded bars) and non assaulted (unshaded bars) physicians in emergency departments**. There was significant difference between two groups as denoted by *P *= 0.01.

**Figure 2:**
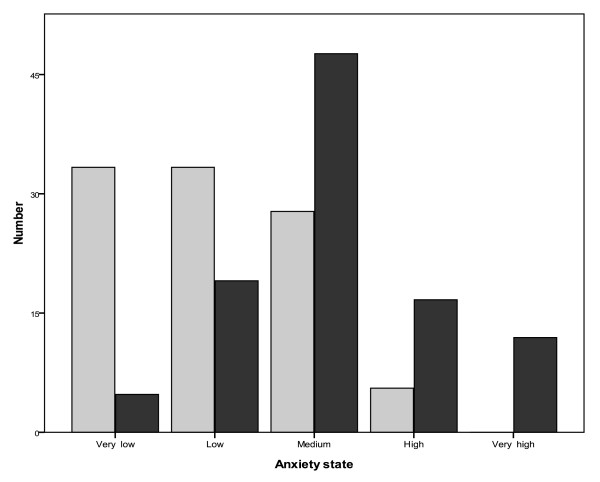
**Comparison of indices of state anxiety inventory between assaulted (shaded bars) and non assaulted (unshaded bars) physicians in emergency departments**. There was significant difference between two groups as denoted by *P *< 0.001.

Cronbach's alpha of the STAI, state, and trait anxiety was respectively 0.88, 0.87, and 0.90. The median [interquartile range, IQR] of SAP between physicians who were victim of violence and those not was significantly different (51[46-59] vs 39 [34-46] respectively; *P *< 0,001). The median [IQR] of TAP between physicians who were victim of violence and those not was also significantly different (48 [41-55] vs 41 [38-43] respectively; *P *= 0,01). Table 2 showed comparison of STAI between groups exposed to violence or not. Figure 1 and 2 showed the comparison of the degree of anxiety state and anxiety trait between assaulted and not assaulted physicians in the emergency departments.

**Table 2:** Comparison of STAI between groups exposed and not exposed to violence

	Assaulted	Not assaulted	*P*
**STAI**	101 (89-109)	77 (69-98)	< 0,001
**Anxiety trait**	48 (41-55)	40 (38-43)	0,01
**Anxiety state**	51 (46-59)	39 (34-46)	< 0,001

STAI = State trait anxiety inventory. *P values are from the chi-squared test, student- test,

or mann-whitney U test to compare the difference between assaulted and non-assaulted

## Discussion

The results of this study show that emergency department physicians are exposed to some form of violence. This study revealed a high prevalence (70%) of violence toward doctors in emergency departments. Verbal abuse appears to be an important risk. The exposure of physicians to some form of violence was related to significant degree of anxiety.

It appears that this international phenomenon is increasing [14,15]. The stark reality is that many aggressive and violent incidents are unreported and so it is likely that this is an under representation of this phenomenon [16]. Aggression and violence may surface in response to a complex multitude of factors [17]. These factors can be grouped into internal (e.g. gender, age, psychiatric illness, drugs and alcohol), external (e.g. overcrowding in wards, lack of space and privacy) and associated factors (e.g. staff and patient interactions) [17]. The effects of aggression and violent behaviour are equally wide and diverse and may negatively impact on staff's physical, psychological, emotional and spiritual health [18]. Factors related to the emergency departments (long waits, high-stress illness, noisy environment and nonselective 24-hour "open-door" policy) may predispose this setting to violence [10]. The reported increase in the frequency and severity of violent incidents over time is not surprising, in view of the increased contact with patients at high risk for initiating violence, such as drug abusers, alcoholics, mentally ill people and gang members [8,19]. Our study found that verbal abuse was most common and physical assaults were experienced less commonly. Verbal and physical violence in the emergency departments are frequent but underreported and have a negative influence on staff working conditions [9]. Schat et al reported recently in their nationally representative probability sample of American workers that 6% of the workforce reported incidents of physical violence over a 12-month period [19]. In contrast, 41.4% of the same respondents reported incidents of psychological aggression. Barling et al concluded that workplace aggression occurs relatively frequently. Workplace violence is an infrequent occurrence [2]. Canbaz et al suggested that the high ratio of verbal abuse may be related to the perception of violence as part of the job. However, the individuals do not dare physical violence because of the laws, which may explain the lower rate of physical assault [7].

Our study included mostly young doctors which cannot predict the predisposition of this population compared to older and therefore more experienced doctors. However, in the social and behavioural sciences younger age is associated with the perpetration of aggression and violence [20]. The possible explanation for this fact is that people understand better the consequences of their behaviour with increasing age, and are more able to exert control over any expression of anger. Studies on the link between age and workplace aggression yield mixed results. Whereas some studies yield a negative correlation between age and workplace aggression [21,22], others yield no significant correlation [5,23,24]. The accident and emergency departments are sometimes considered a hostile environment for junior medical staff [20]. As students and those with less experience are at most risk, Stubbs suggests starting informing future generations that they may experience aggression and violence as part of their undergraduate student programmes [25].

One of the most consistent findings in the social and behavioral sciences is that males tend to be more aggressive and violent than females [26,27]. Our study showed that the aggressor is predominantly male (55%), and this finding was consistent with the results of several studies that showed that males were more concerned by workplace aggression than females [22,24,28,29]. We found a significant relationship link between anxiety trait and workplace violence. Negative affect reflects the individual's predisposition to experiencing negative psychological states such as hostility, sadness, and anxiety. It is subclinical in nature and is differentiated from clinical experiences such as depression [2]. Parkins et al investigated the link between anxiety and workplace bullying and showed no significant relationship [30]. The anxiety regarding repetition of exposure to violence was increased approximately ten-fold in participants who reported having been exposed to violence and was related to higher SAP and TAP [7]. Workplace violence was found to have a negative influence on participants' psychological level, and being responsible for state anxiety is clearly more important. Violence at work increases anxiety. Stress and violence at work are not isolated individual problems, but structural, strategic issues rooted in wider social, economic, organizational and cultural factors [7]. Violence may have negative organizational outcomes in the form of low worker morale, increased job stress, increased worker turnover, and reduced trust of management and co-workers, and a hostile working environment [30]. Under the strain of reforms, growing work pressure and stress, social instability, and the deterioration of personal interrelationships, workplace violence is rapidly spreading in the health sector [7]. Since 1983, Spielberger showed that some individuals are predisposed to respond to what they see as provocation with aggression [31]. Several studies revealed strong correlations between respect and anger and, workplace aggression [23,27,30,32]. Nonetheless, none of these studies have examined these repercussions among physicians especially in the emergency departments. Our study examines the possibility to extrapolate these results to physicians. Our study showed that victim's physicians of violence have already an anxiety trait, and that violence leaves psychological damage as an anxiety state. Nonetheless, our study raises topics for further research, such as comparing the actual incidence and nature of violence to the perception of the respondents, assessing violence prevention programs and measures in the emergency departments; examining the best strategies available to recognize potentially violent situations; and testing strategies to support emergency departments staff who have experienced violence. Would the unique characteristics of the emergency departments necessitate changes in established programs? Addressing this issue may have a beneficial effect on staff well-being, with improved job satisfaction and job retention, reduced fear and better staff-patient relationships.

Our study has several limitations. First, this survey reported only events that occurred in the past 15 days. Second, this is relatively a small study, with 60 emergency physicians responding, this small number represents the number of physicians who have achieved permanence in emergency departments during the 15 days preceding the survey response. The permanence is realized by two physicians in each of medical and surgical units. Third, owing to recall bias, the number of incidents of violence may have been over reported. Fourth, the young age of resident physicians does not allow evaluating the role of age as a predictor, the absence of a comparative population of older age in our study does not allow us to analyze the role of this variable (which is age) as a predicting factor of aggression. Finally, this survey is based on self-reported data and there was no way to verify missing data and the accuracy of data.

## Conclusions

This study revealed a high prevalence (70%) of violence toward doctors in Morocco emergency departments. The exposure of physicians to some form of violence was related to significant degree of anxiety. Efforts should concentrate on the adoption of preventive, systematic and participative interventions. Further research is essential to identify specific risk factors and to describe the epidemiology of aggression and violence toward health care workers that will enable the development of appropriate prevention strategies. This includes:

• Making the reduction/elimination of workplace violence in the health sector an essential part of national and international programs;

• Actively promoting awareness of the risks and destructive impact of workplace violence;

• Providing psychological support to persons exposed to violence.

## Competing interests

The authors declare that they have no competing interests.

## Authors' contributions

JB participated in the design of the study, performed the statistical analysis and draft the manuscript. KB participated in the acquisition of data. FA and AZ participated in the coordination of the study. RA participated in the design of the study, performed interpretation of data, and gave the final approval of the manuscript. All authors read and approved the final manuscript.
